# Glycosyl chains and 25‐hydroxycholesterol contribute to the intracellular transport of amyloid beta (Aβ‐42) in Jurkat T cells

**DOI:** 10.1002/2211-5463.12234

**Published:** 2017-05-18

**Authors:** Neha Sharma, KeangOK Baek, Huong T.T. Phan, Naofumi Shimokawa, Masahiro Takagi

**Affiliations:** ^1^ School of Materials ScienceJapan Advanced Institute of Science and Technology (JAIST)IshikawaJapan; ^2^ Hanoi National University of EducationCaugiayVietnam

**Keywords:** 25‐hydroxycholesterol, amyloid beta‐42, cholera toxin B subunit, intracellular transport, T cells

## Abstract

Amyloid beta (Aβ) is a peptide responsible for the development of Alzheimer's disease (AD). Misfolding and accumulation of endogenous Aβ can lead to neural cell apoptosis through endoplasmic reticulum (ER) stress. Added exogenous Aβ can also result in ER stress, leading to neurotoxicity and apoptosis, which is identical to that caused by the endogenous peptide. We have speculated that the endocytic transport of Aβ causes ER stress and have previously shown that the oxysterol, in particular, 7‐ketocholesterol (7‐keto) induces more surface interaction between Aβ‐42 and Jurkat cells than cholesterol. However, the interaction was not enough to induce intracellular transfer of the peptide. In this study, we investigated the effect of another oxysterol, 25‐hydroxycholesterol (25‐OH) on the membrane raft‐dependent transport of Aβ‐42 in Jurkat cells. Interestingly, intracellular transfer of Aβ‐42 was observed in the presence of 25‐OH only after the inclusion of cholera toxin B subunit (CT‐B), a marker used to detect the raft domain. We speculated that 25‐OH can induce intracellular movement of Aβ peptides. Furthermore, CT‐B together with GM1 provided negative curvature, which resulted in the intracellular transport of Aβ‐42. Notably, we used a protofibrillar species of Aβ‐42 in this study. We have shown that the transport was microtubule‐dependent since it could not be observed in depolymerized microtubules. These results demonstrate that oxysterols and glycosyl chains are important factors affecting intracellular transport. These compounds are also associated with aging and advanced glycation are risk factors for AD. Thus, this study should further understanding of the pathology of AD.

**Abbreviations**
24S‐OH: 24‐S‐hydroxycholesterol
25‐OH: 25‐hydroxycholesterol
7‐keto: 7‐ketocholesterol
Aβ: amyloid beta
AD: Alzheimer's disease
AGE: advanced glycation end‐products
AMD: age‐related macular degeneration
APP: amyloid precursor protein
BACE: beta‐site APP cleaving enzyme
CNS: central nervous system
CT‐B: cholera toxin B subunit
ER: endoplasmic reticulum
GM1: monosialoganglioside
LXRα: liver X receptor α
ROS: reactive oxygen species
SM: sphingomyelin

Aggregation and accumulation of amyloid beta (Aβ) is the pathological pathway of the neurological disorder commonly known as Alzheimer's disease (AD). Amyloid precursor protein (APP) is a transmembrane protein (Type 1) that undergoes different catabolic pathways at three main cleavage sites. Although APP is present in every tissue, it is most highly expressed in the brain. It is believed to be the precursor protein for Aβ, produced following cleavage of APP via beta site APP cleaving enzyme (BACE) and γ‐secretase. Endoplasmic reticulum (ER) is responsible for correctly folding or processing newly synthesized proteins, and when proteins like Aβ accumulate in the ER in misfolded form, it can cause stress to the organelle resulting in cellular apoptosis. This phenomenon is known as ER stress. According to previous reports, externally added Aβ can cause ER stress in primary neurons 1, 2, 3 on addition of oligomeric species of Aβ‐42. We speculated that this could be due to the endocytic vesicular transport of Aβ. ER stress also occurs when the homeostasis of ER is disturbed, which is a process associated with other neurodegenerative diseases and diabetes. Diabetes is linked to AD and is a known risk factor. Other risk factors that increase the chance of AD include aging, advanced glycation end‐products (AGEs), and genetic and metabolic abnormalities. For example, aging increases the prospect of AD development via the production of reactive oxygen species (ROS), which cause the oxidation of lipids. In our study, we considered these factors and explored the roles of each in the intracellular and/or endocytic movement of Aβ peptides.

Recent studies have suggested different mechanisms for the internalization of Aβ, such as endocytic mechanisms, the pore‐forming protein perforin, and receptor‐mediated pathways 4. Endocytic transport or endocytosis is a membrane phenomenon and the basic structure of the membrane is a lipid bilayer, which is largely composed of cholesterol, sphingolipids, and glycerophospholipids 5. Most cholesterol (25%) in the human body is present in the brain 6 and is oxidized enzymatically and nonenzymatically to produce side chain‐oxygenated derivatives with an additional hydroxyl group, such as 24‐S‐hydroxycholesterol (24S‐OH), 25‐hydroxycholesterol (25‐OH), and 27‐hydroxycholesterol (27‐OH), or an extra oxygen group following ROS‐induced oxidation to produce 7‐ketocholesterol (7‐keto), 7β‐hydroxycholesterol (7β‐OH), etc. Recent reports have demonstrated the importance of oxysterols in AD progression due to altered metabolism of cholesterol and oxidative stress 7. Cholesterol metabolism is controlled by 24S‐OH and 25‐OH through binding to liver X receptor α (LXR α). Yamanaka *et al*. 8 found that 24S‐OH causes necroptosis of neural cells in a concentration‐dependent manner.

We performed a comparative study on the interaction of the peptide with biomimetic membranes while studying peptide Aβ using its two isoforms, Aβ‐40 and Aβ‐42 9, 10, 11, 12. We previously reported that the inclusion of 7‐keto results in greater surface interaction between protofibrillar Aβ‐42 and Jurkat T cells 11, although peptide transport was not observed. The accumulation of Aβ is suggested to be higher in cholesterol‐poor domains in biomimetic and biological membranes 9, 11 and higher thermosensitivity is induced by 25‐OH compared with cholesterol and 7‐keto in lipid membranes 10. Furthermore, 25‐OH has been shown to be more cytotoxic compared with other oxysterols such as 7β‐OH, 7‐keto, 19‐hydroxycholesterol (19‐OH), 22‐R‐hydroxycholesterol (22(R)‐OH), and 22‐S‐hydroxycholesterol (22(S)‐OH) 13, inducing cytotoxicity in various cell types 13, 14, 15. Consistent levels of 25‐OH have been found in mouse brain along with other oxysterols 16. It is also reported that 25‐OH opens the mitochondrial permeability transition pore and increases the production of ROS, resulting in the release of cytochrome *c* and further activation of caspase‐3 and ‐9 14.

Thus, for the present study, we chose 25‐OH to investigate the role of oxysterols in the intracellular movement of Aβ‐42 in Jurkat T cells, and compared the effects with cholesterol and 7‐keto. Among the two isoforms, we used Aβ‐42 because it is more hydrophobic and toxic than Aβ‐40. We have largely used protofibrillar (elongated oligomers) species to date, which are considered to be the primary toxic forms of Aβ‐42 along with oligomers 17.

In the current study, we used Jurkat T cells (a line of T cells). They are reported to be a target of Aβ 18 and express a certain level of caspase‐8, which is a key mediator of the extrinsic apoptotic pathway 19. To achieve our aim, we studied cellular toxicity induced by the peptides, membrane‐peptide interaction, changes in intracellular calcium levels, and the mechanistic pathway of proteins in cells with added 25‐OH. The results obtained in this study will provide a basis for our understanding of the mechanisms involved in AD.

## Materials and methods

### Materials

25‐hydroxycholesterol, bovine serum albumin (BSA), and trypan blue were obtained from Sigma‐Aldrich (St. Louis, MO, USA). Aβ protein (human, 1–42; Aβ‐42) was obtained from Peptide Institute Inc. (Osaka, Japan), HiLyte FluorTM 488‐labeled (λex = 503 nm, λem = 528 nm) Aβ‐42 and HiLyte Fluor‐555‐labeled (λex = 551 nm, λem = 567 nm) Aβ‐42 were obtained from Anaspec, Inc. (Fremont, CA, USA). ER‐Tracker Blue‐White DPX, Oregon Green 488 taxol, Roswell Park Memorial Institute 1640 (RPMI1640) medium, FBS, and Alexa Fluor 555‐conjugated cholera toxin B subunit (CT‐B; λex = 555 nm, λem = 565 nm) were obtained from Invitrogen (Eugene, OR, USA). Phosphate buffer salts (PBS), and Tris (hydroxymethyl) aminomethane (Tris) were purchased from Takara Bio Inc. (Shiga, Japan) and Kanto‐Chemical (Tokyo, Japan), respectively.

### Cell culture

Jurkat human leukemic T cells (Riken Cell Bank, Ibaraki, Japan) were cultured in medium containing RPMI 1640 supplemented with 10% FBS in a humidified incubator at 37 °C in the presence of 5% CO_2_.

### Preparation of protofibrillar Aβ‐42

Two hundred micromolars of Aβ‐42 solutions were prepared by dissolving Aβ‐42 powder in 0.02% (v/v) ammonia solution and stored at −80 °C. Prior to the experiments, Aβ‐42 solution was diluted to a concentration of 80 μm with Tris buffer (20 mm, pH 7.4), and subsequently incubated at 37 °C for 12 h 10. To prepare the protofibrillar species, fluorescence‐labeled Aβ‐42 and normal Aβ‐42 were mixed at a 1 : 2 molar ratio before incubation 9.

### Lipid raft staining

Membrane cholesterol levels were altered by treating the cells with 25‐OH in PBS at two different concentrations, 5 and 10 μm, for 10 min at 37 °C. Lipid rafts were labeled by treating cells with 1 μg·mL^−1^ Alexa fluor 555‐labeled CT‐B, which specifically binds to monosialoganglioside (GM1) in lipid rafts 20, and 0.02% (v/v) BSA in PBS at 0 °C for 30 min, followed by incubation at 37 °C for 10 min. Cells were subsequently washed with PBS buffer.

### Measurement of cell viability

Trypan blue exclusion assay was used to estimate Jurkat cell viability upon the addition of Aβ‐42 protofibrils 21, 22. Control cells and added with 25‐OH cultured in a 48‐well plate were exposed to 10 μm protofibrillar Aβ‐42 for 24 h. Cells were then washed with PBS and treated with trypan blue at a final concentration of 0.01% (v/v) in PBS for 10 min at room temperature. The numbers of dead and viable cells were counted using a haemocytometer. Cells with clear cytoplasm were defined as viable, while dead cells had blue cytoplasm 23. Cell viability was calculated following the format: cell viability = total viable cells/total cells. Data are expressed as means ± SD of at least three independent experiments.

### Staining and depolymerization of microtubules

Oregon Green^®^ (Invitrogen) 488 Taxol was used to stain the microtubules. Cells were treated with 20 μg·mL^−1^ nocodazole for 10 min at 37 °C in a humidified incubator in the presence of 5% CO_2_ to achieve depolymerization. Thereafter, cells were washed twice with PBS.

### Staining of endoplasmic reticulum

Endoplasmic reticulum was labeled by treating cells with 1 μg·mL^−1^ ER‐Tracker (Blue‐White DPX dye) in 0.02% BSA, followed by incubation at 37 °C for 30 min. Cells were subsequently washed with PBS buffer.

### Measurement of intracellular calcium level

Intracellular calcium levels were measured using a visible Ca^2+^ fluorescent probe, Fluo‐3‐AM 24. Fluo‐3‐AM is an acetoxymethyl ester derivative of Fluo‐3, which forms complexes with calcium after hydrolysis by nonspecific esterase in the cell. Control, 25‐OH added cells were cultured in 48‐well plates and subsequently exposed to 10 μm protofibrillar Aβ‐42 for 60 min 25. Cells were then washed with RPMI 1640 (nonserum) and loaded with 4 μm Fluo‐3‐AM with 20% (w/v) pluronic F127 with PBS at 37 °C for 20 min. 0.01% (w/v) BSA in PBS was added to dilute the cells five‐fold, and plates were incubated further for 40 min. Probe‐loaded cells were washed and resuspended in HEPES buffer. Calcium levels were measured at an excitation of 488 nm 26 using confocal laser scanning microscopy. In addition, calcium levels were measured using Flow‐cytometer (Beckmann Quanta SC, Beckmann Coulter, Brea, CA, USA). We measured the Fluo‐3‐AM intensity of intracellular space. The intracellular Ca^2+^ level is defined by the fluorescent intensity of Fluo‐3‐AM based on that for cell without Aβ‐42 and 25‐OH.

### Microscopy

Fluorescence‐labeled Aβ‐42 was added to T‐cell suspension at a final concentration of 5 μm. The resultant mixture was placed in a glass bottom dish and observed using a confocal laser scanning microscope (FV‐1000; Olympus, Tokyo, Japan), 60× Lens, at room temperature within 2 min. The observation period was short enough to avoid the effect of fluorescent quenching 10.

### Statistical analysis

To process images, Olympus Fluoview and image j software were used. Images are one representative experiment of three performed. Data are expressed as means ± SD of three independent experiments. Comparisons between different membranes were performed using *t*‐test and ANOVA test wherever applicable.

## Results

### Incorporation of 25‐OH in the cell membrane

**Figure 1:**
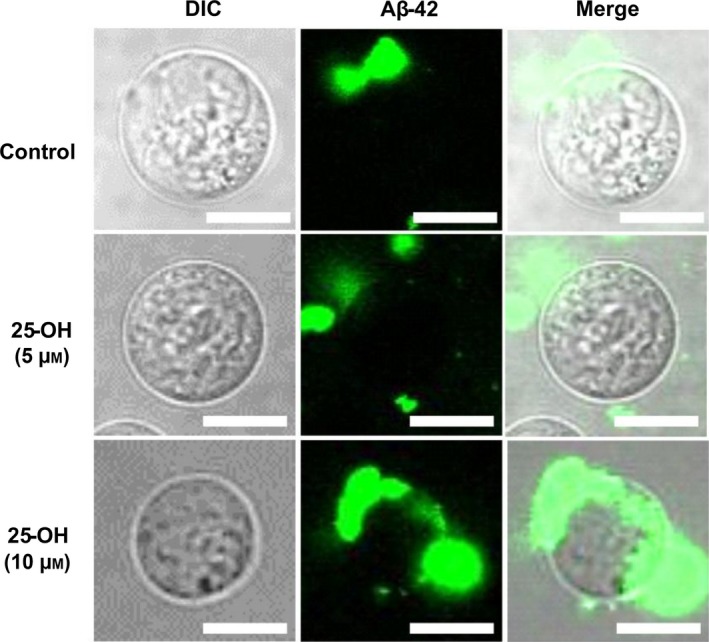
Addition of 25‐OH and exposure to protofibrillar Aβ‐42. Representative microscopic images of Jurkat T cells both control (basal level cholesterol) and with added 25‐OH (5 and 10 μm) after exposure to protofibrillar Aβ‐42. Gray image is DIC microscopic image of the cell, green fluorescence represents HiLyte Fluor‐labeled Aβ‐42 (488 nm) taken by confocal laser scanning microscopy and merged images show overlapping of the two forms. Images of one representative experiment out of three performed. Number of cells observed were 150–200. Scale bars = 10 μm.

We have previously shown that the interaction between Aβ‐42 and cells is enhanced after inclusion of 7‐keto in the cell membrane 11. Our previous study compared the effects of basal level cholesterol, excessive additional cholesterol, and additional 7‐keto in cells. Intracellular transport of the peptides could not be observed in any of these cases. Thus, we investigated the interaction between Aβ‐42 and Jurkat T cells in the presence of another oxysterol, 25‐OH. Cells with basal cholesterol levels taken as control and those added with 25‐OH were observed at two different concentrations, that is, 5 and 10 μm, and single‐cell observation could be achieved via confocal laser scanning microscopy. In Fig. 1, gray image is the differential interference contrast image (DIC) of the cell while green fluorescence is of fluorescence species of Aβ‐42. Merged images clearly prove the accumulation of Aβ on the surface of the cell membrane. Accumulation of Aβ‐42 was observed in 25‐OH added cells as well. However, intracellular transport of peptides was not induced in this system.

### Involvement of GM1 in endocytic transfer of Aβ‐42 in Jurkat T Cells

**Figure 2:**
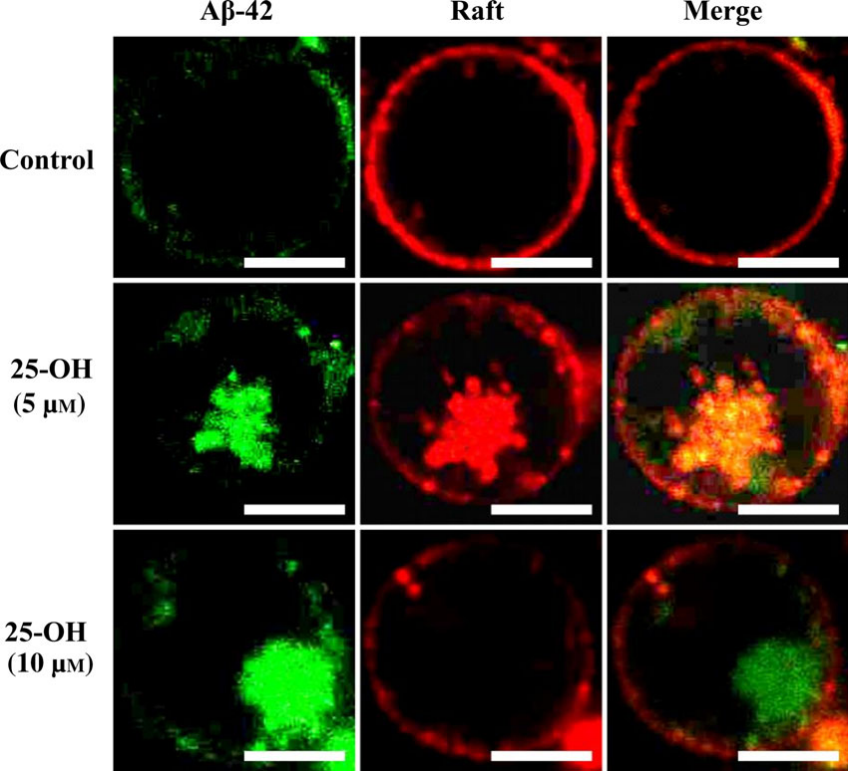
Role of CT‐B and 25‐OH in intracellular transport of protofibrillar Aβ‐42. Representative confocal microscopic images of Jurkat T cells both control and with added 25‐OH (5 and 10 μm). Green and red fluorescence represent HiLyte Fluor‐labeled Aβ‐42 (488 nm) and CT‐B (555 nm), respectively. Images of one representative experiment out of three performed. Number of cells observed were 150–200. Scale bars = 10 μm.

We previously reported that the interaction between GM1 and CT‐B facilitates endocytic movement and transformation in the membrane 27. Thus, CT‐B, which binds to GM1 was used to highlight the lipid raft. We then observed cells at basal cholesterol level and with 25‐OH at 5 and 10 μm in the presence of CT‐B, and expose them to Aβ‐42. In Fig. 2, green and red fluorescence represents Aβ‐42 and lipid raft, respectively. At basal cholesterol level, Aβ‐42 accumulates at the surface of cell membrane only. As mentioned earlier, in 7‐keto‐added cells, transport of Aβ‐42 was not observed at 5 and 10 μm. Therefore, we considered that 7‐keto added cells could be the negative control for the present study 11. However, in 25‐OH added cells, Aβ‐42 appeared inside the cells. Merged images show the colocalization of raft and Aβ‐42. We observed the intracellular transport of Aβ‐42 after the inclusion of CT‐B and additional 25‐OH in the cells. CT‐B appeared essential for the transport of peptide because Aβ‐42 was not internalized into the membrane in its absence as explained above. Furthermore, GM1 and CT‐B induces negative curvature in model membranes 27, which probably resulted in the transport of Aβ‐42 in Jurkat cells. At 5 μm 25‐OH, Aβ‐42 was internalized and colocalized with the raft, thus this process was named ‘raft‐dependent transport’. Previously, Aβ‐42 peptides are reported to internalize via raft‐dependent endocytosis in neuroblastoma cells 28. However, at 10 μm, Aβ‐42 appeared without the raft, hence we named this process ‘raft‐independent transport’. This mode of transport of the peptides is not a well‐known process, and we need to clarify the precise reasons.

### Effect of oxysterols and Aβ‐42 on cell viability

**Figure 3:**
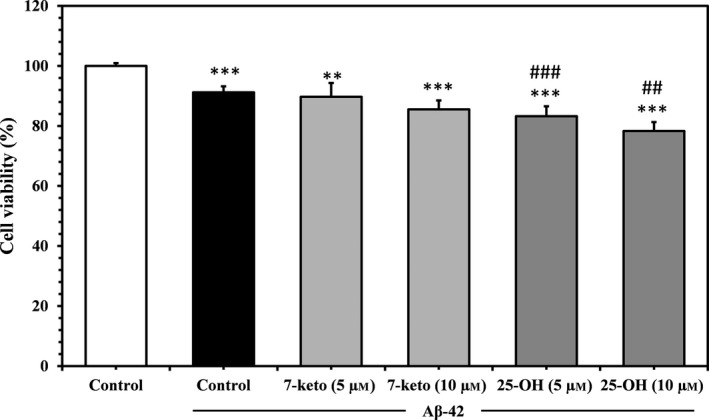
Effect of oxysterols on Aβ‐induced toxicity on Jurkat T cells. Cell viability of Jurkat T cells: control, with added 7‐keto and 25‐OH (5 and 10 μm) after exposure to protofibrillar Aβ‐42 for 24 h. Viability was measured using Trypan blue exclusion assay as described in the text. The values are means ± SD of three independent set of identical experiments. The symbols **, *** indicate significant differences between the cells exposed to protofibrillar Aβ‐42 and the untreated cells in the absence of the peptide (*P* ≤ 0.01 and *P* ≤ 0.001), respectively). The symbols ##, ### indicate a significant difference between oxysterol‐added cells and the untreated cells in response to protofibrillar Aβ‐42 (*P* ≤ 0.5 and *P* ≤ 0.01, respectively).

To measure the effects of toxicity induced by Aβ‐42 in oxysterol‐added cells, cell viability was measured using Trypan blue exclusion test. Cells were treated with protofibrillar Aβ‐42 (10 μm) for 24 h, which caused a mild decrease in cell viability. Substitution in the composition of the membrane with addition of 25‐OH at 5 and 10 μm and exposure to Aβ‐42 caused ~ 17% and 22% depression, respectively, in cell viability. The effect of 25‐OH was > 7‐keto at similar concentrations and incubation times (Fig. 3). Although the inclusion of oxysterol and exposure to Aβ‐42 is harmful to cells, it had few effects under the specified conditions in Jurkat cells. It is very important to consider that independent effect of oxysterols on the cell viability was diminished under the used conditions since pretreatment of cells with oxysterols was performed for 10 min. Similar concentrations of 25‐OH are lethal for neural cells 13, 14 and will kill ~ 35–40% of cells but after 48 h treatment. In addition, these concentrations of oxysterols were employed to avoid cytotoxicity to study about the role of the same in endocytic mechanisms of Aβ peptides.

### Role of microtubules in the transfer of Aβ‐42

**Figure 4:**
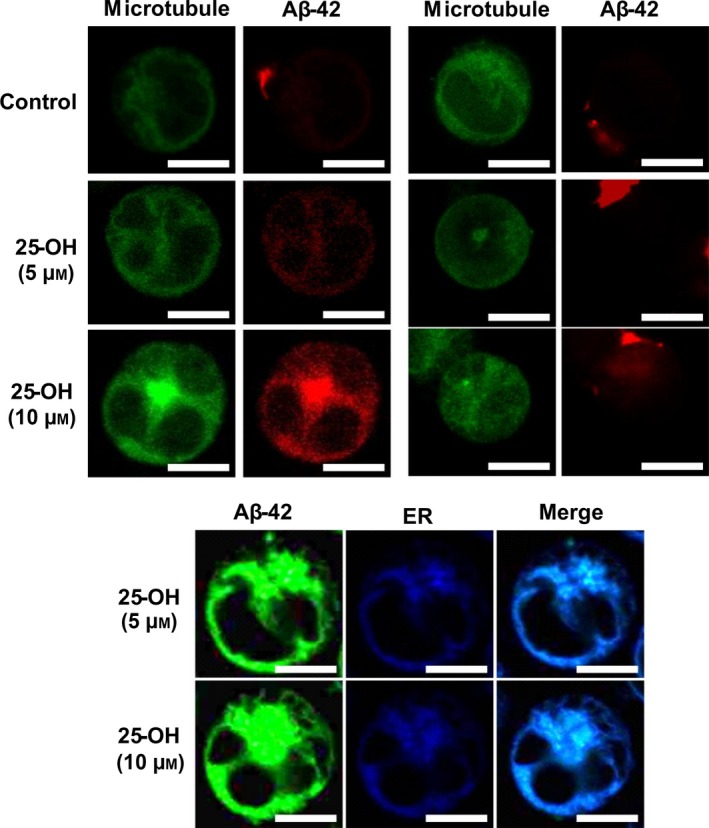
Pathway of intracellular trafficking of Aβ‐42 and its localization in ER. Representative confocal microscopic images of Jurkat T cells both control and with added 25‐OH (5 and 10 μm) after exposure to protofibrillar Aβ‐42. Observation of microtubules were performed in its polymerized and depolymerized microtubule state. Green and red fluorescence represents Oregon green (488 nm) and HiLyte Fluor‐labeled Aβ‐42 (555 nm), respectively. Observation of Aβ‐42 in the ER was observed in the provided images. Green and blue fluorescence represents HiLyte Fluor‐labeled Aβ‐42 (488 nm) and ER (350–460 nm), respectively. Images of one representative experiment out of three performed. Number of cells observed were 150–200. Scale bars = 10 μm.

The cytoskeleton is a crucial component of cells and is comprised of three parts, one of which is known to be the microtubule, which has the longest filament. After intracellular transport of Aβ‐42, it must follow a certain pathway to move to the ER. We used Oregon green to stain the microtubules and depolymerized them via nocodazole, a depolymerizing agent. When microtubules were in polymerized state, in control cells, Aβ‐42 could not be observed inside the cells, as shown in Fig. 4. Conversely, Aβ‐42 appeared inside the cells along with microtubules in the presence of 25‐OH. Afterwards, in a depolymerized state, Aβ‐42 could be observed at the cell surface in control cells as well as in 25‐OH added cells at both its concentrations (5 and 10 μm). Hence, it is possible that Aβ‐42 internalizes and reaches the ER via microtubules. It was suggested that the delivery of proteins to their destinations is achieved via microtubules, sorting signals, and motor proteins in neural cells. Thus, microtubules carry Aβ peptides from the vicinity of plasma membrane to its destination which can be ER, mitochondria, Golgi bodies, or other cell organelle. Lana *et al*. 4 demonstrated that Aβ‐42 colocalizes with ER, lysosome, and mitochondria of differentiated SH‐SY5Y cells. Since in the depolymerized state of microtubules, Aβ‐42 resided only at the surface of membrane, thus depolymerization of microtubules possibly disturbs the endocytic transport of the peptides.

### Transfer of Aβ‐42 to endoplasmic reticulum

Endoplasmic reticulum stress is a result of the advancement of misfolded proteins in the ER. To check whether internalized Aβ would reach to ER, we stained the organelle with ER tracker. As can be seen in Fig. 4, Aβ‐42 was colocalized with ER. It is known that exogenous Aβ causes ER stress which can cause cell death and is accompanied by the release of Ca^2+^ since the ER is a calcium reservoir.

### Intracellular calcium release in oxysterol‐added cells

Intracellular Ca^2+^ levels were measured before and after the inclusion of oxysterols, and after exposure to protofibrillar Aβ‐42 for 1 h 25. Fluo‐3‐AM probe was used which is an indicator of intracellular Ca^2+^. Intensity of the probe represents the number of calcium ions which binds with dye. The release of Ca^2+^ could be a result of ER stress since the ER is a calcium reservoir, and altered homeostasis leads to efflux of Ca^2+^.

**Figure 5:**
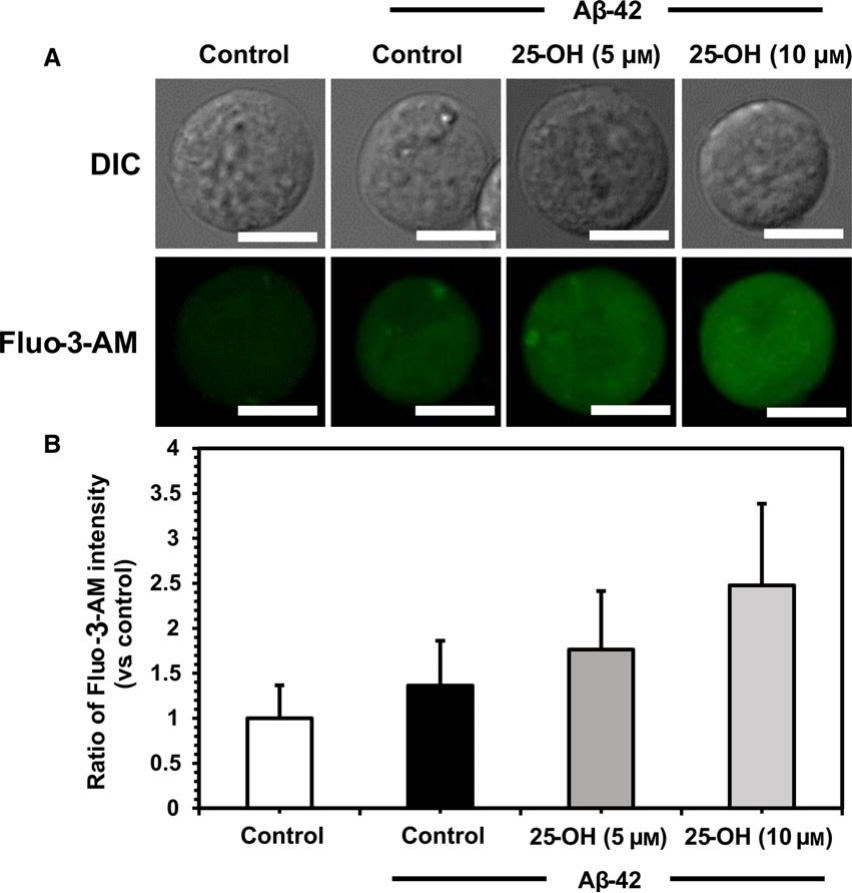
Changes in intracellular calcium level. (A) Microscopic images of both control and with added 25‐OH cells with and without exposure to protofibrillar Aβ‐42 for 1 h and subsequently loaded with Fluo‐3‐AM to measure the release of calcium. Upper and lower images were taken by DIC microscopy and confocal laser scanning microscopy, respectively. Green fluorescent represents Fluo‐3‐AM (488 nm). (B) Graphical representation of the effects of 25‐OH (5 and 10 μm) on intracellular calcium release represented by the fluorescent intensity of Fluo‐3‐AM based on that for cell without Aβ‐42 and 25‐OH. Images of one representative experiment out of three performed. Values are given as means ± SD of three independently performed experiments. Number of cells observed were 150–200. Scale bars = 10 μm.

We observed a remarkable increase in calcium levels after the addition of 25‐OH and exposure to Aβ‐42 in Jurkat cells (Fig. 5). In comparison with 7‐keto, 25‐OH caused a greater release of ions (data not shown); thus, a more profound effect than that of 7‐keto. In addition, we have not considered the effect of oxysterols by themselves since our intention was to observe the changes occurred in the activity of Aβ‐42 on Ca^2+^ release in the presence of oxysterols. Moreover, incubation period of oxysterols with the cells was short enough to avoid significant changes caused by oxysterols as reported earlier 10, 11. On the other hand, there are alternative sources or reservoirs of Ca^2+^, such as mitochondria, etc. Even if this increase in Ca^2+^ may not be a direct indication of ER stress, but it signifies the Aβ‐induced perturbations in the cell.

## Discussion

Oxysterols are derivatives of cholesterol and considered to have substantial biological effects. The roles of the different oxysterols, such as 7‐keto, 24S‐OH, 22‐OH, and 7β‐OH in AD have been studied in the past and have been attributed to the modulation of cell permeability 29, 30, regulation of cholesterol homeostasis 31, 32, regulation of gene expression 33, 34, and they act as receptors in cell signaling 35, 36. 27‐OH and 24S‐OH are both reported to be involved in AD 8, 37 and age‐related macular degeneration (AMD) 38, and 25‐OH shares structural similarities with both oxysterols. Furthermore, it has been suggested that 25‐OH interposes the transcription factors related to the synthesis of cholesterol and fatty acids 39.

Herein, we focused on the need for 25‐OH in the intracellular transport of Aβ‐42 in Jurkat cells. Jurkat line is a potential candidate for the study of AD because the plasma membrane of these cells is rich in sphingomyelin (SM) and has functional and structural diversity in GM1‐rich domains 40. The use of Jurkat T cells gave us the opportunity to deepen our knowledge of the essential factors involved in endocytic mechanisms. We changed the composition of the membrane by adding 25‐OH and compared the data with results obtained from the inclusion of excess cholesterol and 7‐keto in Jurkat T cells. Excessive cholesterol causes higher accumulation of Aβ‐42 on the cell surface 11, but compared with oxysterols, the addition of cholesterol resulted in weaker interaction between Aβ‐42 and the cells and alterations via 7‐keto led to stronger surface interaction. Internalization of protofibrillar Aβ‐42 was observed in the presence of 25‐OH in Jurkat cells. Furthermore, previously we had demonstrated 7‐keto could not cause the intracellular movement of Aβ‐42 even together with GM1 and CT‐B. Thus, 7‐keto‐added cells were considered as the negative control for the 25‐OH‐added cell system. From this previous result and our present results, 25‐OH, Aβ‐42, GM1, and CT‐B are needed for the achievement of transport of Aβ‐42 in the cells. Since membrane deformation such as endocytosis occurs using excess surface area generally, the acquisition of excess surface area becomes an important factor. It was reported that 25‐OH causes excess surface area increase in the Aβ‐induced membrane which was higher than increase observed in the presence of 7‐keto 10. Therefore, we can observe the intracellular transport in 25‐OH‐added membranes, however, it did not occur in 7‐keto‐added membranes due to smaller excess surface area. In addition, to select the endocytic transport as membrane deformation, the induction of negative curvature is essential. This has been already reported that the interaction between GM1 and CT‐B leads to the induction of negative curvature in model membranes 27. Therefore, the intracellular transport of Aβ‐42 is achieved by these two important factors, acquisition of large excess surface area and induction of negative curvature.

Cholera toxin B subunit binds with GM1, which is a widely distributed sphingolipid present in all types of tissue, but is in highest concentrations in the central nervous system (CNS). GM1 clusters are reported to bind to Aβ peptides and accelerate the formation of toxic Aβ fibrils 41. Ample evidence suggests that membrane lipids have an integral role in the accumulation and formation of Aβ fibrils 42, which accumulate in GM1‐rich regions and are also highly concentrated in the CNS. Therefore, we can speculate that the internalization of Aβ fibrils may become crucial in the CNS. These facts strengthen the theories surrounding the onset of AD, which is a neurological disorder.

We checked the viability of cells after exposure to Aβ‐42 and oxysterols. Although the changes induced by 7‐keto and 25‐OH are not significant in Jurkat T cells, 25‐OH led to a greater reduction in cell viability than 7‐keto (Fig. 3). Previously, 25‐OH was found to stimulate apoptosis in oligodendrocytes and the expression of secreted phospholipase enzyme (sPLA_2_‐IIA) 43. 25‐OH decreased the viability of neuronal PC12 cells to 61% after 3 days of treatment 44. Although, oxysterols have been reported to induce apoptosis in lymphoma cells (RDM4) at 5 and 10 μm after 48 h treatment 45. But, experimental conditions and concentration of oxysterols used in our study, were not enough to induce the depression in the cell viability by the individual action of oxysterols.

After observing the internalization of Aβ‐42, we were interested to learn about the pathway used by the peptides for transport into the cell. Thus, we analyzed the cytoskeleton to determine the mode of transport. Internalization of Aβ‐42 can occur in the polymerized state, thus nocodazole, a chemical used to depolarize microtubules which was used to evaluate whether internalization is cytoskeleton dependent or independent. Usually, microtubules stabilize the structure of a cell and are organized by tau protein, which is another protein involved in AD; disruption of the microtubules is a fundamental feature in AD patients although the primary mechanism is still unknown.

Colocalization of Aβ‐42 and ER indicates that Aβ‐42 reaches the ER after internalization within the cell. Compared with cholesterol, 25‐OH swiftly transports from the cell membrane to the ER 46. Intracellular calcium increases after the addition of 25‐OH in a concentration‐dependent manner, thus we speculated that this was caused by Ca^2+^ released from the ER. A mild increase in Ca^2+^ release is observed with 24S‐OH, and it is thought that it is released from the cytoplasm following 24S‐OH‐induced toxicity 47. 25‐OH elevates calcium permeability in biological membranes 48, 49 and, presumably, permeability results from the movement of 25‐OH between the lipid bilayers 50. In addition, 25‐OH has been found to produce toxicity in aortic smooth muscle cells via a calcium‐dependent mechanism after the treatment of cells with 25‐OH alone for a period of 24 and 48 h 51. Moreover, we had used Jurkat cells for our study where 25‐OH at specified conditions could not produce stress by itself. According to previous reports using Jurkat cells and 25‐OH confirms that presence of oxysterol will enforce the calcium ionophore to increase mRNA levels at 0.5 μg·mL^−1^ with 10 μg·mL^−1^ cholesterol for 48 h 52. It indicates that calcium homeostasis can be disturbed by 25‐OH after almost 2 days of treatment in Jurkat cells.

To summarize the findings of our study, we wanted to emphasize the risk factors of AD. Although there are many known risk factors for AD but here we focused precisely on role of oxysterols and glycation. According to generally accepted facts, AD is an old age dementia, and aging is one of the primary factors that lead to ROS. ROS cause the formation of lipid peroxidation products, specifically cholesterol derivatives (oxysterols) and oxysterols are known as potential markers in neurological disorders. We have previously shown that 7‐keto causes greater surface interaction and have demonstrated that 25‐OH assists in the intracellular transport of Aβ peptides. Thus, oxysterols themselves emerges as risk factors in AD.

Other than aging, changes in the structure and function of proteins could cause accumulation and misfolding. These changes in proteins can be achieved by a process known as glycation where reducing sugars form advanced glycation end‐products (AGE). *In vitro* studies have shown that glycated Aβ peptides tend to aggregate faster than nonglycated forms. Consequently, the size of the aggregated species is larger, and these forms stabilized Aβ peptides. These aggregated and misfolded peptides cause cell dysfunction and apoptosis. Receptor for advanced glycation end‐products (RAGE) is closely associated with the endocytic transfer of Aβ peptides. Furthermore, it is highly expressed in the brains of AD patients. Aβ is a major cause of AD, conformation of the peptide is closely related to the induction of toxicity by Aβ. Oligomers and protofibrils species of Aβ have been proposed as the primary toxic species of the peptide. Thus, aggregated species of Aβ plays integral role in the toxicity caused by the peptide, they are correlated with endocytic transfer enhanced by oxysterols and glycation.

The production and accumulation of AGE species are also involved in metabolic conditions, such as diabetes, which is a known risk factor in the development of AD. Diabetes is caused by glycation of insulin and insulin is an essential growth factor present in the brain. Thus, lack of glucose regulation may contribute to high blood sugar levels in the brain and damage to blood vessels, thus diabetes is relevant to neurological disorders such as AD and Parkinson disease. Monomeric forms of insulin promote amyloid aggregation. Similarly, glycosyl chains of GM1 that bind to Aβ peptides, are reported to induce the growth of Aβ. Internalization of Aβ in neural cells is relevant to the abundant presence of GM1, which comprises 6% of total phospholipids in the nervous system. In our study, the GM1‐CT‐B interaction induced negative curvature and intracellular transport of peptides.

Advanced glycation end‐products species also correlate with aging and the production of ROS, and it has been found that ribosylated amyloid aggregates cause a significant increase in the amount of ROS species and cell death on exposure to cells 53. These proteins are detected at a higher rate in elderly people and are therefore a prominent factor in age‐related diseases such as diabetes and AD. Moreover, aging, oxidative stress, and the production of ROS and AGE species are integral contributors to the pathology of diabetes and AD.

Thus, our experimental data support the fact that oxysterols and glycosyl chain interactions are risk factors in AD. We have demonstrated the importance of these factors in the endocytic transport of Aβ peptides, a cause of ER stress. To further our understanding on the mechanism behind the endocytic transport of Aβ, our next step will be to use human neuroblastoma SH‐SY5Y cell line to deepen our knowledge of the role of oxysterols and glycation in the progression of AD. Further study may concern to know about the factors involved in the endocytic transport of peptide, concentration dependency of the oxysterols, and their independent effect on cell viability and other changes produced which may leads to AD.

## Conclusions

We have demonstrated some important factors for the intracellular transport of the Aβ‐42 using Jurkat cell line, which is an optimized model for understanding the behavior of neural cells in AD. Notably, we used a toxic isoform of Aβ peptide. Our study was a comparative study using different oxysterols to alter the composition of the membrane. The use of CT‐B was proposed when the addition of oxysterols was not sufficient for the intracellular transport of the peptide. GM1 interaction with CT‐B provided a negative curvature, which helped prove, in a model membrane, that endocytosis is an important factor in living cells. Thus, 25‐OH and the GM1‐CT‐B interaction are considered vital for the internalization of peptides inside the cell. Internalization was proven to occur via the microtubules (cytoskeleton) of the cell. Thereafter, Aβ‐42 was localized in the ER, suggesting that the final destination of the peptide is the ER. The roles of these oxysterols in neurodegenerative studies of diseases such as AD will further our understanding of dementia in elderly people.

## Author contributions

NS, MT, and KB designed the experiments. NS performed the experiments and wrote the manuscript under the guidance of rest of the authors. NS critically reviewed the manuscript and provided substantial support in the research. HTTP provided an intellectual input and help in designing the experiments. MT coordinated the study. All authors have approved the final version of the manuscript.
